# HPF Modulates the Differentiation of BMSCs into HLCs and Promotes the Recovery of Acute Liver Injury in Mice

**DOI:** 10.3390/ijms24065686

**Published:** 2023-03-16

**Authors:** Bin Yang, Qiu-Lin Luo, Nan Wang, Yan-Ting Hu, Wen-Xin Zheng, Hong Li, Maimaitituxun Maierziya, Jian Gu, Qin Wang

**Affiliations:** 1 School of Pharmacy, Southwest Minzu University, Chengdu 610225, China; binyang1109@163.com (B.Y.); qiulin1104@163.com (Q.-L.L.); 17711380471@163.com (N.W.); h18185425548@163.com (Y.-T.H.); zhengwenxin1124@163.com (W.-X.Z.); lh19970099@163.com (H.L.); m18224067141@163.com (M.M.); 2 BMI Center for Biomass Materials and Nanointerfaces, College of Biomass Science and Engineering, Sichuan University, Chengdu 610065, China

**Keywords:** herpetfluorenone, bone marrow-derived mesenchymal stem cells, hepatocyte-like cells, alleviate acute liver injury

## Abstract

Bone marrow-derived mesenchymal stem cells (BMSCs) can differentiate into hepatocyte-like cells (HLCs) to alleviate acute liver injury (ALI). Herpetfluorenone (HPF), as an active ingredient in the dried, mature seeds *Herpetospermum caudigerum* Wall, used in Tibetan medicine, has been proven to effectively alleviate ALI. Therefore, the purpose of this study was to determine whether HPF can promote the differentiation of BMSCs into HLCs and promote ALI recovery. Mouse BMSCs were isolated, and the BMSCs’ differentiation into HLCs was induced by HPF and hepatocyte growth factor (HGF). Under the induction of HPF and HGF, the expression of hepatocellular specific markers and the accumulation of glycogen and lipids in the BMSCs increased, indicating that BMSCs successfully differentiated into HLCs. Then, the ALI mouse model was established, using carbon tetrachloride, followed by an intravenous injection of BMSCs. Then, only HPF was injected intraperitoneally, in order to verify the effect of HPF in vivo. In vivo imaging was used to detect the homing ability of HPF–BMSCs, and it was detected that HPF–BMSCs significantly increased the levels of serum AST, ALT and ALP in the liver of ALI mice, and alleviated liver cell necrosis, oxidative stress and liver pathology. In conclusion, HPF can promote the differentiation of BMSCs into HLCs and promote the recovery of ALI in mice.

## 1. Introduction

The liver has antibacterial, antiviral and drug detoxification properties, and is essential for maintaining physiological balance [1]. ALI is one of the main causes of liver diseases, with high morbidity and mortality [2]. ALI is usually caused by virus infection, drug or alcohol abuse, and the ingestion of toxic substances [3,4].The mechanism of ALI is mainly related to the interaction of hepatocyte degeneration, inflammatory reaction, reactive oxygen species, hepatocyte necrosis and apoptosis [5]. Severe ALI may lead to cirrhosis, liver failure and death, but there is no effective targeted drug available at present. In general, orthotopic liver transplantation is considered the most effective treatment [6]. However, the shortage of liver donors, the high cost of operation and the rejection of transplantation restrict the clinical application of orthotopic liver transplantation. Therefore, we should develop more effective and non-transplanted drugs to treat ALI.

According to research findings, stem cells can regenerate and repair damaged liver cells [7,8]. Among them, BMSCs have shown their therapeutic potential in liver diseases [9]. BMSCs have a higher reproductive capacity, lower immunogenicity and the ability to self-renew. When compared with embryonic stem cells, BMSCs are easier to obtain and have no major ethical or religious concerns. Therefore, BMSCs have been explored to treat liver diseases, such as liver injury, liver fibrosis, cirrhosis and liver failure [9,10,11,12]. However, the ability of BMSCs to transplant the target tissue has been proven to be unsatisfactory, thus influencing their therapeutic effect. In addition, it has been widely proven that BMSCs can directly differentiate into HLCs in vitro [13,14]. More importantly, HLC transplantation can be utilized as an effective therapy for ALI. Therefore, it is imperative to search for potential drugs that can promote the differentiation of BMSCs into HLCs, so as to improve the repair effect of ALI.

HGF is a pleiotropic cytokine with significant mitogenic activity and can induce hepatocyte proliferation during liver regeneration [15]. It has been reported that HGF can induce BMSCs to differentiate into mature HLCs. However, it is expensive and difficult to obtain. It is necessary to find new drugs to improve the existing induction methods. Numerous studies have shown that compound medicinal preparations, single herbs, and herb extracts in traditional Chinese medicine (TCM) have specific roles in regulating the proliferation and differentiation of BMSCs. By exploring the effects of TCM on the differentiation of BMSCs, the application of BMSCs in liver tissue engineering can be improved and broadened [16,17]. HPF is an active monomer component, isolated from the total lignans of dried mature seeds, found in *Herpetospermum caudigerum* Wall, a plant used in Tibetan medicine, which has a broad spectrum of biological activities, including anti-fatigue, anoxia-resistant, and antiviral activities [18]. Meanwhile, HPF has significant protective effects on liver diseases, such as anti-hepatitis B virus, anti-tumor activity and ALI [18]. However, it is unclear as to whether HPF can effectively promote the differentiation of BMSCs into HLCs and the recovery of ALI in mice.

Therefore, in this study, we first isolated mouse BMSCs and verified that HPF can enhance the ability of HGF to induce BMSCs to differentiate into HLCs. Then, carbon tetrachloride was used to further establish the ALI mouse model, and HPF was verified to promote the differentiation of BMSCs into HLCs and to promote the recovery of ALI in vivo.

## 2. Results

### 2.1. Culture and Characterization of Rat BMSCs

**Figure 1:**
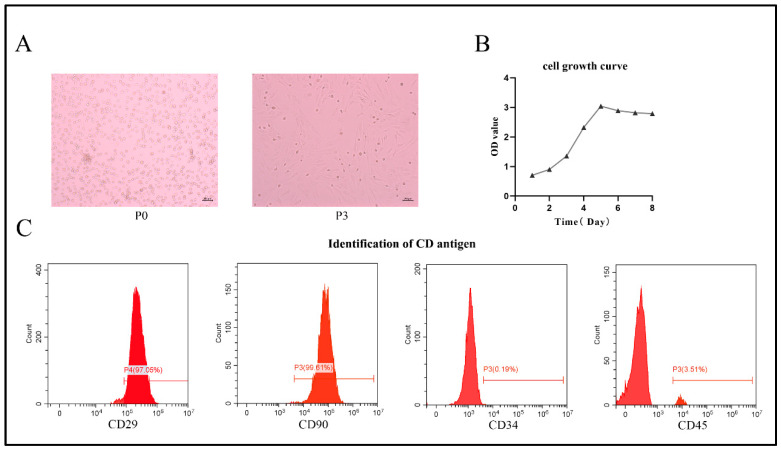
Morphological characteristics of BMSCs. (**A**) Morphological characteristics of BMSCs at P0 and P3 under the microscope (50× magnification). (**B**) CCK-8 staining, demonstrated the proliferative capability of BMSCs. (**C**) Flow cytometry analysis for CD29, CD90, CD34 and CD45 expression, to identify BMSCs.

Freshly isolated BMSCs appeared spherical in terms of their morphology (Figure 1A, left). Incubated for 5 to 7 days, BMSCs reached 80% confluence and were passaged. After three passages, the cells became more uniform and exhibited a long spindle type (Figure 1A, right). Using CCK-8 data, the growth curve of BMSCs showed an “S” growth pattern, indicating that BMSCs have a strong ability to proliferate (Figure 1B). Flow cytometry showed high expression levels of CD29 and CD90, but low expression levels of CD34 and CD45. The percentages are as follows: CD29, 97.05%; CD90, 99.61%; CD34, 0.19%; CD45, 3.51% (Figure 1C). The morphological characteristics of BMSCs and the expression of relevant cell surface markers were compatible with the criteria proposed by the International Society for Cellular Therapy (ISCT) [19]. Therefore, we confirm that the main cell population extracted from bone marrow comprises BMSCs.

### 2.2. Identification of HPF

**Figure 2:**
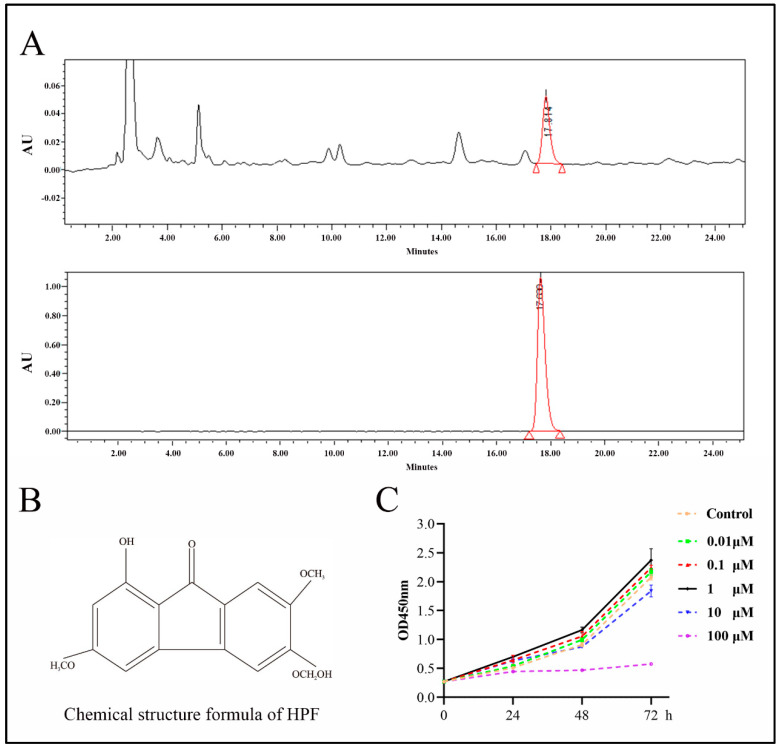
The chemical composition of HPF was determined by high-performance liquid chromatography (HPLC). (**A**) HPLC chromatogram of Herpetspermumb and HPLC chromatogram of HPF standard. (**B**) Chemical structural formula of HPF. (**C**) OD value distribution of HPF with different concentrations.

The compound HPF was identified by comparing the peak retention time of a single peak with that of a real reference standard (Figure 2A,B). Subsequently, the cell proliferation experiment showed that HPF concentrations of 1 μmol/L, 0.1 μmol/L and 0.01 μmol/L had proliferation effects on BMSCs, and the proliferation effect of HPF at 1 μmol/L was the most significant. Therefore, this concentration of HPF was used for subsequent experiments (Figure 2C).

### 2.3. HPF Can Accelerate the Differentiation of Rat BMSCs

**Figure 3:**
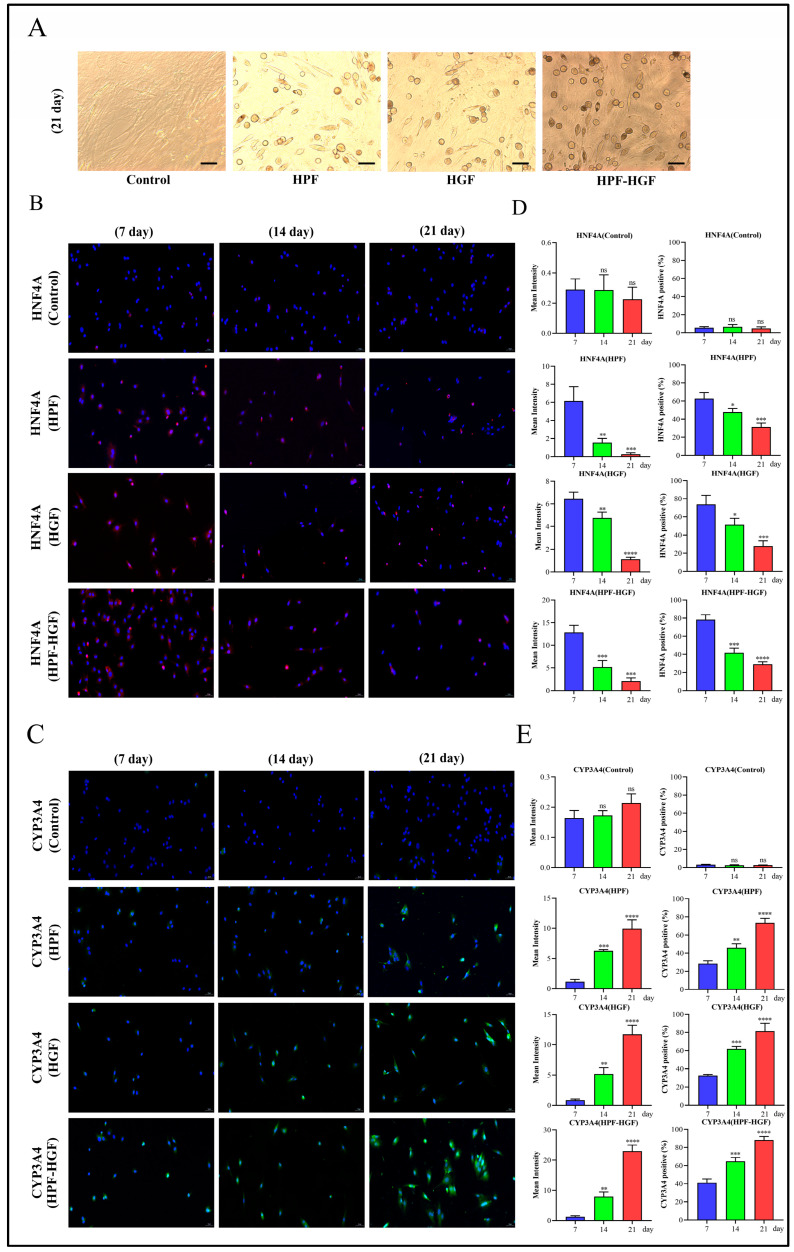
Inducing BMSCs to differentiate into HLCs. (**A**) Morphological observation of the differentiation of BMSCs into HLCs, stimulated by HPF, HGF and HPF–HGF for 21 days (200× magnification). (**B**,**C**) The expression of the hepatocyte markers HNF4A and CYP3A4 in HPF-, HGF- and HPF–HGF-treated BMSCs, as well as in untreated BMSCs, was detected by immunofluorescence staining (HNF4A in red, CYP3A4 in green, DAPI-stained nuclei in blue) (200× magnification). (**D**,**E**) The immunofluorescence staining results of HNF4A and CYP3A4 were analyzed by mean fluorescence intensity and positive rate. * *p* < 0.05, ** *p* < 0.01, *** *p* < 0.001, **** *p* < 0.0001 and ns (no statistical significance), compared with the group treated for 7 days.

**Figure 4:**
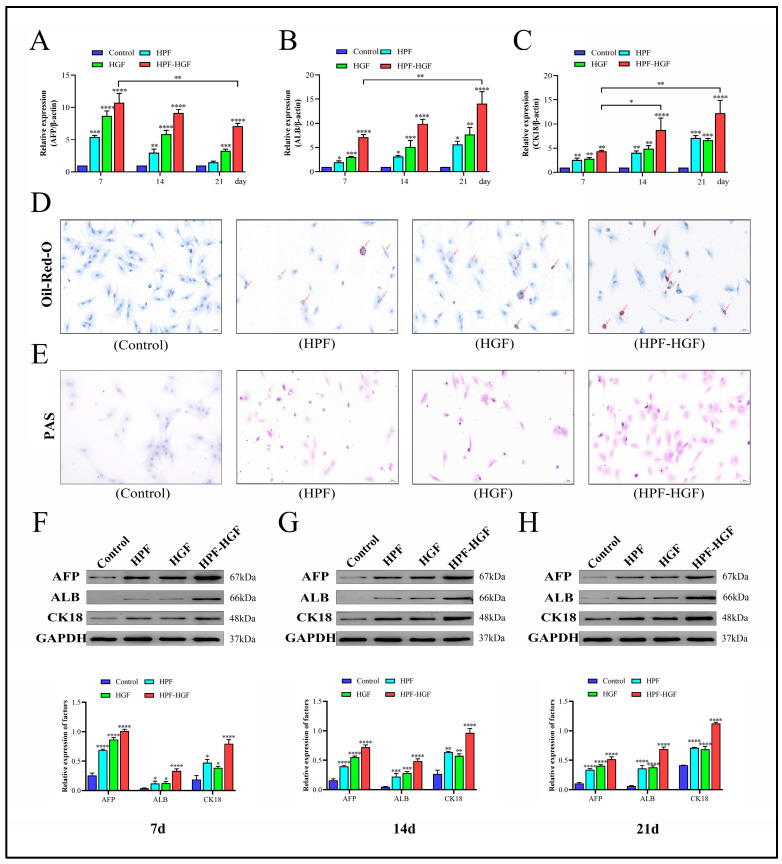
Inducing BMSCs to differentiate into HLCs. (**A**) RT-qPCR was used to detect the expression of AFP in HPF-, HGF- and HPF–HGF-treated BMSCs and untreated BMSCs, at different time points. (**B**) RT-qPCR was used to detect the expression of ALB in HPF-, HGF- and HPF–HGF-treated BMSCs and untreated BMSCs at different time points. (**C**) RT-qPCR was used to detect the expression of CK18 in HPF-, HGF- and HPF–HGF-treated BMSCs and untreated BMSCs at different time points. (**D**) Representative images of BMSCs stained with Oil-Red-O show the ability of BMSCs to store lipids at 21 days of differentiation (200× magnification). (**E**) Representative images of BMSCs stained with PAS show the ability of BMSCs to store glycogen at 21 days of differentiation (200× magnification). (**F**) Expression of the hepatocyte markers AFP, ALB and CK18 in BMSCs treated with HPF, HGF and HPF–HGF for 7 days, as well as in untreated BMSCs, was detected by Western blot. (**G**) Expression of the hepatocyte markers AFP, ALB and CK18 in BMSCs treated with HPF, HGF and HPF–HGF for 14 days, as well as in untreated BMSCs, was detected by Western blot. (**H**) Expression of hepatocyte markers AFP, ALB and CK18 in BMSCs treated with HPF, HGF and HPF–HGF for 21 days, as well as untreated BMSCs, was detected by Western blot. * *p* < 0.05, ** *p* < 0.01, *** *p* < 0.001 and **** *p* < 0.0001 compared with the control group.

In order to study the effect of HPF on the differentiation of BMSCs, HPF and HGF were added to BMSCs, and cells were collected after 7, 14 and 21 days of culture. The morphological characteristics of the control group, HPF group, HGF group and HPF–HGF group after 21 days of differentiation were observed under a fluorescence microscope. The results showed that the control group showed a typical fibroblast-like morphology, and the HPF, HGF and HPF–HGF groups showed circular, oval and polygonal morphologies, respectively, similar to that of hepatocytes, and significantly different from BMSCs. (Figure 3A). Subsequently, immunofluorescence staining was used to analyze the expression of the liver precursor cell marker HNF4A and liver cell marker CYP3A4 at days 7, 14 and 21 (Figure 3B,C). We further analyzed the mean fluorescence intensity and positive rates of HNF4A and CYP3A4, and the results showed that there was no difference in the expression of HNF4A and CYP3A4 in untreated BMSCs (Figure 3D,E). The expression of HNF4A in the HPF, HGF and HPF–HGF groups was the highest on the seventh day of differentiation, decreased on the fourteenth day, and had decreased even more on the twenty-first day, with statistically significant differences (Figure 3D). In addition, CYP3A4 expression in the HPF, HGF and HPF–HGF groups was the lowest on the seventh day of differentiation, increased on the fourteenth day, and reached the highest levels on the twenty-first day, with statistically significant differences (Figure 3E). Our results showed that HPF, HGF and HPF–HGF induced BMSCs all expressed HNF4A and CYP3A4, and the effect of HPF–HGF was superior to that of HPF and HGF alone. Meanwhile, the expression of hepatocyte-related markers (alpha-fetoprotein (AFP), albumin (ALB), and cytokeratin 18 (CK18)) in BMSCs was detected by reverse transcription quantitative polymerase chain reaction (RT-QPCR). We found that AFP, ALB, and CK18 were almost not expressed at all in BMSCs without HPF and HGF induction; these genes were expressed in both HPF- and HGF-induced BMSCs, and the expressions of the combined-induction BMSCs were greater than those of the two single-use BMSCs (Figure 4A–C). Oil-red-O staining showed that more oil could be detected in HPF- and HGF-induced BMSCs, while Oil-Red-O staining was negative for BMSCs without them (Figure 4D). Periodic acid Schiff (PAS) staining showed higher glycogen content in HPF- and HGF- induced BMSCs, while BMSCs without HPF and HGF addition were negative for PAS staining (Figure 4E). In addition, protein Western blotting was used to analyze the expression of AFP-, ALB-, and CK18-associated markers in liver cells. We found that AFP, ALB, and CK18 were minimally expressed or virtually non-expressed in untreated BMSCs, while the BMSCs supplemented with HPF and HGF showed significantly increased expressions of AFP, ALB, and CK18 (Figure 4F–H). Collectively, BMSCs can induce HPF and HGF to differentiate into HLCs, and the combination of HPF and HGF induction can increase the differentiation effect of HLCs.

### 2.4. HPF Combined with BMSCs in the Treatment of ALI in Mice

**Figure 5:**
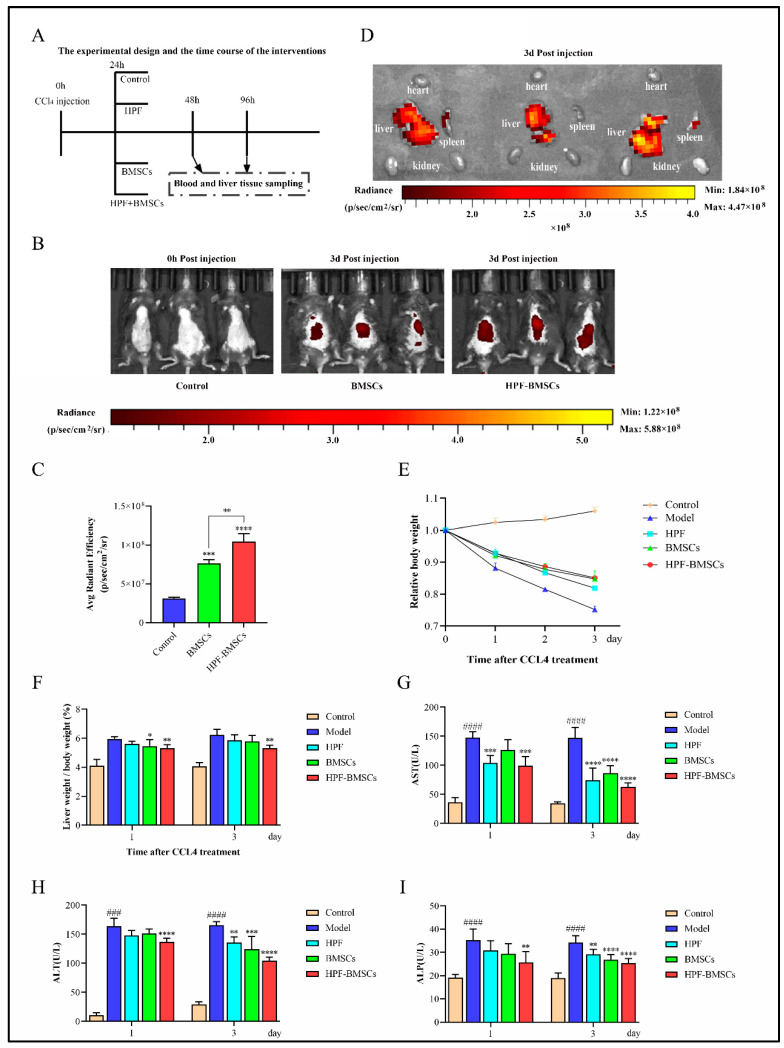
HPF combined with BMSCs improves CCl_4_-induced ALI in mice. (**A**) Time flow chart of experimental design and intervention. (**B**) Fluorescence display of blank control group, BMSC group and HPF–BMSC group on the 3rd day after BMSC injection. (**C**) Statistics of fluorescence intensity, representing BMSCs’ homing to the liver. (**D**) Fluorescence distribution of the HPF–BMSC group in the main organs, on the 3rd day after injection of BMSCs. (**E**) Relative body weight. (**F**) Liver index. Serum levels of (**G**) AST, (**H**) ALT and (**I**) ALP. ### *p* < 0.001 and #### *p* < 0.0001 compared with control group, * *p* < 0.05, ** *p* < 0.01, *** *p* < 0.001 and **** *p* < 0.0001 compared with the model group.

To evaluate the role of HPF combined with BMSCs, in the treatment of ALI in mice induced by acute liver injury, ALI was successfully induced by an intraperitoneal injection of CCl_4_ in mice. The schematic diagram of the experimental process is shown in Figure 5A. Next, 5 × 10^5^ BMSCs stained with DiD were injected into the modeled mice with ALI through the caudal vein for a single time, and the distribution of fluorescence imaging in the BMSC group and HPF–BMSC group was observed on the third day after the injection of DiD-BMSCs. The results showed that when DiD-BMSCs were injected through the tail vein on the third day, the fluorescence imaging areas in vivo were mainly concentrated near the liver and spleen. When compared with the control group and BMSC group, the average fluorescence intensity of the HPF–BMSC group is much higher (Figure 5B,C). At the same time that the the fluorescence distribution in the main organs of the HPF–BMSC group was observed on the third day of DiD-BMSCs injection, fluorescence imaging was performed in all the major organs of mice (heart, liver, spleen, and kidney), and the results showed that the fluorescence was mainly concentrated in the liver, while the fluorescence intensity of the other organs was either negligible or very low (Figure 5D). The results showed that the intraperitoneal injection of HPF could promote the homing ability of BMSCs. Next, we analyzed the livers of ALI mice. Weight loss and liver index increases might indicate liver damage [3]. We recorded and counted the body weight of mice during the experiment. The results showed that, when compared with the control group, the body weight of mice in the model group decreased significantly, while the body weight of mice in each administration group increased to different degrees (Figure 5E). The liver functions of mice were further assessed. The levels of liver indexes and serum biomarkers (AST, ALT, and ALP) in mice are shown in Figure 5F–I. When compared with the control group, the above indexes were significantly increased in the model group, indicating that CCl_4_ successfully induced ALI in mice. However, the HPF, BMSC, and HPF–BMSC groups significantly and dose-dependently attenuated the increase in biomarker levels in the model group. These results indicate that HPF and BMSCs can promote healing of the liver injury induced by CCl_4_, as well as enhance the therapeutic effect of HPF. The BMSC group performed significantly better than groups treated with HPF and BMSCs alone.

### 2.5. Liver Morphology and HE Staining

**Figure 6:**
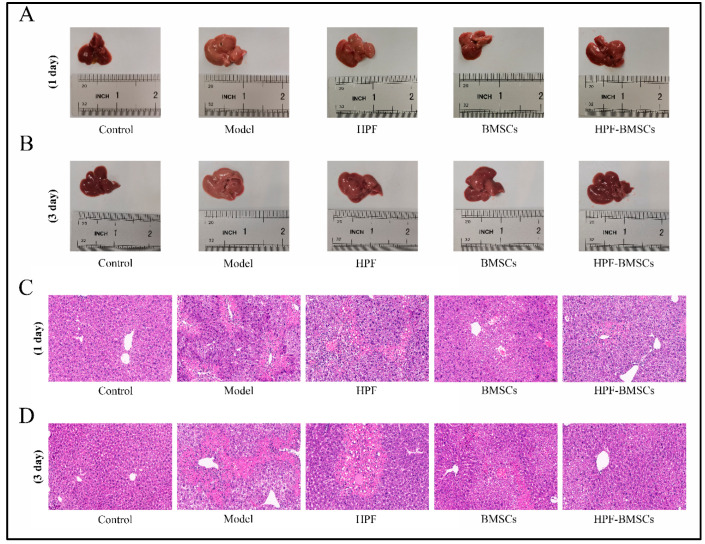
HPF combined with BMSC treatment improved hepatic pathological changes in mice with CCl_4_-induced liver injury. (**A**,**B**) Morphology of the liver tissues in each group. (**C**,**D**) The representative images of HE staining. Scale bars: 50 μm.

As seen in Figure 6A,B, in the control group, the liver was bright red, and the membrane surface was smooth and soft. When compared with the control group, the liver volume of the model group was significantly increased, the surface of the capsule was rough, the texture was hard, local rot was visible, and the color was dark red. When compared with the model group, the liver volume of each administration group was reduced slightly, the liver edge was slightly pointed, the surface was slightly rough, the texture was slightly hard, and the color was slightly bright red. In addition, HE staining results of the liver are shown in Figure 6C,D. In the control group, the liver lobule structure was normal, the liver cord was arranged neatly, the liver cells were intact, the cytoplasm was colored evenly, and there was no obvious inflammation, cell degeneration or necrosis. In the model group, the hepatic cord was disordered, the lobular structure was mostly destroyed, and vacuolar degeneration of surrounding liver tissue, inflammatory cell infiltration and bleeding were observed. Hepatic cord disorders, unclear boundaries of hepatic lobules, obvious degeneration and necrosis of hepatic cells, moderate diffuse vacuolar degeneration, and light inflammatory infiltration in the HPF group were observed. In the HPF–BMSC group, the hepatic cord was mildly disordered, the structure of the hepatic lobule was easily seen, the liver cells were mildly denatured, the inflammatory infiltration was significantly reduced, and the pathological manifestations of liver tissue tended to be normal. The pathological changes in the BMSC group comprised an intermediary stage between the above two groups. The results indicated that the treatment effect when ALI was induced 3 days after administration was better than the treatment effect when ALI was induced 1 day after administration. Therefore, mice subjected to acute liver injury 3 days after treatment administration were used for follow-up experiments.

### 2.6. Effect of HPF-BMSCs on Expression Level of Oxidative Stress

**Figure 7:**
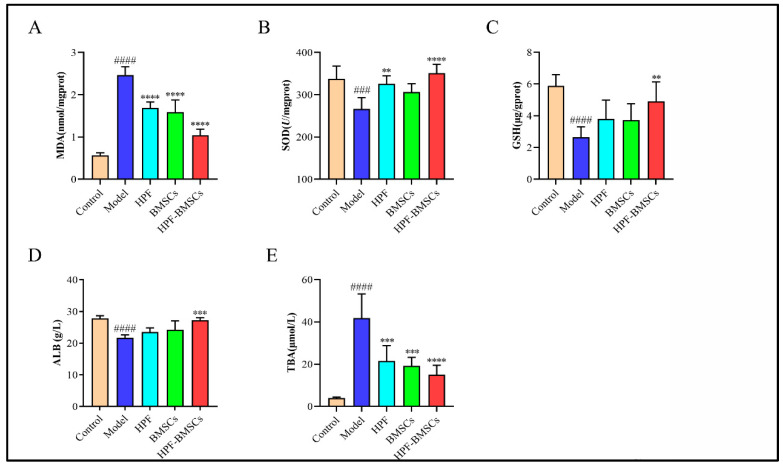
The combined treatment of HPF and BMSCs changed the liver antioxidant enzyme activity of mice with ALI, induced by CCl_4_. The activities of (**A**) MDA, (**B**) SOD, (**C**) GSH, (**D**) ALB, and (**E**) TBA. ### *p* < 0.001 and #### *p* < 0.0001 compared with control group, ** *p* < 0.01, *** *p* < 0.001 and **** *p* < 0.0001 compared with the model group.

The activity of antioxidant enzymes (MDA, SOD and GSH) in the mouse liver is shown in Figure 7A–C. When compared with the control group, the activity of SOD and GSH in the liver tissue of the model group was significantly decreased, and the activity of MDA was significantly increased, indicating that the liver cells were damaged. Relative to the model group, the SOD and GSH activities of liver tissue in the HPF, BMSC and HPF–BMSC groups were significantly increased, while MDA levels were significantly decreased. These findings suggest that HPF and BMSCs may reduce the oxidative stress produced by CCl_4_.

### 2.7. Hepatic Levels of ALB and TBA

As shown in Figure 7D,F, the contents of ALB and TBA in the liver were determined. The results showed that, compared with the control group, the content of ALB in the model group was significantly lower, while the content of TBA was greatly higher. When compared with the model group, the content of ALB increased but the content of TBA decreased in each treatment group. These data confirmed that the combination of HPF and BMSCs can alleviate the degree of liver cell injury and promote the regeneration of liver cells, thus achieving the effect of treating ALI in mice.

## 3. Discussion

This study explored the potential of HPF-induced differentiation of BMSCs into HLCs, as well as how this process would prevent liver injury and promoted liver regeneration in mice with CCl_4_-induced ALI. The novel findings of this study are summarized as follows: (1) it was proven, for the first time, that HPF has the potential to promote the differentiation of BMSCs into HLCs, while the combination of HPF and HGF can significantly improve the differentiation efficiency of BMSCs; (2) HPF significantly improved the homing efficiency of BMSCs in the injured liver; (3) after treatment with BMSCs and HPF, the liver function and the morphology of the liver injury were improved considerably; (4) after treatment with BMSCs and HPF, the symptoms of ALI were vastly reduced.

Liver regeneration has always been of great therapeutic significance, especially regarding the generation of HLCs from other types of cells (adult stem cells and induced pluripotent stem cells). This cell type can be used to create cell-based therapies, study the mechanism of human disease and human development, and provide a platform for pharmacological and toxicological drug screening [20]. It is reported that ALB, CK18 and AFP are markers of hepatocytes [21]. HLCs have some functional liver activities, such as the secretion of urea, the storage of glycogen, and the accumulation of neutral triglycerides and lipids in the cytoplasm [22]. ALB, AFP and CK18 are widely recognized as hepatocyte-specific genes [23]. In addition, previous studies showed that after HGF induced BMSCs to differentiate into HLCs, the expression of ALB and CK18 was up-regulated, and the content of glycogen was increased [14,24,25]. In vitro, the combination of HPF and HGF can accelerate the differentiation of BMSCs to HLCs. The results showed that these HLCs had typical a hepatocyte morphology and unique hepatocyte markers, such as ALB, AFP, CK18, HNF4A, and CYP3A4. At the same time, HLCs also show liver functions, such as glycogen storage and fat accumulation. The above research results are partially consistent with our results. Therefore, we can conclude that HPF is an inducible small molecule that promotes the differentiation of BMSCs into HLCs.

CCl_4_ is a recognized hepatotoxin that can attack and damage liver cells. At present, CCl_4_ has been widely used in the experimental model of liver injury, and is one of the classic methods used to inducing ALI [26,27]. Its mechanism of action mainly involves oxidative stress, inflammatory reaction, cell apoptosis, and liver cell necrosis [3]. AST, ALT, and ALP are mainly distributed in the mitochondria and cytoplasm. When the liver is damaged, the permeability of hepatocyte membrane increases, and AST, ALT, and ALP leak into the blood, resulting in a sharp increase in their content. Therefore, serum AST, ALT, and ALP are recognized as biomarkers of liver injury. In addition, liver index is also considered as an important index to evaluate the degree of liver injury [28]. In an in vivo experiment, the intraperitoneal injection of CCl_4_ resulted in a significant increase in the liver index and serum levels of AST, ALT and ALP levels, suggesting that the ALI model was successful. However, the addition of HPF, BMSCs and HPF–BMSCs reduced the increase of these indicators. In particular, the effect of the HPF–BMSC group is the most obvious, indicating that HPF and BMSCs can treat ALI by restoring liver function. These findings were also confirmed by the observation of liver morphology and HE staining.

It is worth noting that the histopathological examination shows that HPF–BMSCs have therapeutic effects in restoring the liver structure. HE staining and the elevation of ALB levels confirm that HPF and BMSCs can promote the repair of damaged liver and the regeneration of hepatocytes. The effect of the HPF group and BMSC group on improving ALI is not as good as that of the HPF–BMSC group, which may be due to the synergistic promotion of HPF and BMSCs.

Our research still has some limitations. The molecular pathway behind the anti-ALI effect of HPF–BMSCs has not been elucidated. Thus, further in-depth study is needed.

In conclusion, HPF combined with HGF can promote the ability of BMSCs to differentiate into HLCs. In addition, HPF can significantly improve the recovery of biochemical markers in severely damaged liver tissue and serum by promoting the homing of BMSCs to the damaged liver, and ultimately promote the recovery of ALI in mice. These findings provide a preliminary conceptual proof that the combination of HPF and transplanted BMSCs is feasible and effective, and the treatment of ALI in mouse models has therapeutic benefits. Therefore, the combination of HPF and BMSCs can be used as a new treatment for ALI.

## 4. Materials and Methods

### 4.1. Materials and Reagents

HPF (>98% purity) was purchased from Chengdu ConBon Biotech Co., Ltd. (Chengdu, China). Alanine aminotransferase (ALT), aspartate aminotransferase (AST), alkaline phosphatase (ALP), SOD, GSH, MDA, ALB and TBA test kits were obtained from Nanjing Jiancheng Bioengineering Institute (Nanjing, China). CCl_4_ was provided by Shanghai MACKLIN Biochemical Technology Co., Ltd. (Shanghai, China). PAS and Oil-Red-O were provided by Beijing Solarbio Science Tenchnology Co., Ltd. (Beijing, China). hepatocyte growth factor (HGF) was provided by PeproTech (Rocky Hill, NJ, USA).

The following antibodies were utilized in this study: AFP (ab213328, Abcam, London, UK), CK18 (ab133263, Abcam, London, UK), HNF4A (ab199431, Abcam, London, UK), ALB (16475-1-AP, Proteintech, Chicago, IL, USA), GAPDH (10494-1-AP, Proteintech, Chicago, IL, USA), and CYP3A4 (1742R, Bioss, Beijing, China).

### 4.2. Isolation and Culture of Rat BMSCs

BMSCs were obtained from the bone marrow of male SD rats (2–3 weeks of age) by adherent centrifugation. First, SD rats were killed by cervical dislocation and soaked in 75% alcohol for 15 min. Then, the femur and tibia were excised carefully and cut into the metaphysis. The marrow cavity was repeatedly rinsed with complete medium (85% DMEM low sugar, 15% fetal bovine serum, 1% 100 U/mL penicillin and 100 mg/mL streptomycin) until it turned white. Bone-marrow-isolated cells were centrifuged for 5 min, and the sediments were re-suspended with complete medium. The cells were cultured in a cell incubator (37 °C, 5%CO_2_) and the medium was changed every 3 days. When the cells fused to 70–80%, the passage operation could then be performed. To minimize variability, third-generation BMSCs were used in all liver differentiation studies.

### 4.3. Basic Characteristics of BMSCs

In order to detect cell morphology, BMSCs were examined under an electron microscope after the third passage.

The third-generation BMSCs were cultured for cell proliferation tests, and the growth degree of BMSCs was measured and evaluated by cell counting kit-8 (CCK-8) for 7 consecutive days. BMSCs were planted in 96-well plates (3000/well) and kept alive. Then, 10 μL of CCK-8 solution was added to each well, and BMSCs were incubated at 37 °C for 2 h. The absorbance of each well was measured at 450 nm with an enzyme-labeled meter.

### 4.4. Flow Cytometry

BMSCs of the third generation were removed from the medium, and then rinsed twice with 1 × 10^5^ cells/mL PBS. Cells in PBS were stained with 5 μL fluorescein isothiocyanate (FITC)- or phycoerythrin (PE)-conjugated antibodies: CD29, CD90, CD34 and CD45. Flow cytometry was used for washing, and surface markers on BMSCs were analyzed.

### 4.5. The Effect of HPF on BMSCs Proliferation Was Determined by the CCK-8 Method

HPF with transcriptional activity was selected and dissolved with DOSO, in order to make mother liquor. BMSCs cultured to the logarithmic growth stage were inoculated into 96-well plates at the rate of 5 × 10^3^/well, and were cultured overnight in an incubator. The blank group and the experimental group were set up respectively. The blank group was added with DMSO as the solvent control, and the experimental group was added with the final concentrations of 0.01 μmol/L, 0.1 μmol/L, 1 μmol/L, 10 μmol/L and 100 μmol/L HPF with transcriptional activity, with five parallel replications in each group. After 24, 48 and 72 h of treatment, CCK-8 solution, an amount that was 10% of the total volume, was added to each well. The solution was incubated in the incubator for 2 h, and the OD_450_ was determined by enzymoleter.

### 4.6. Inducing Liver Differentiation

The third-generation BMSCs were inoculated in 6-well plates and incubated in basal differentiation DMEM (high glucose), supplemented with 10% FBS (Gibco), 100 U/mL penicillin G and 100 μg/mL streptomycin. BMSCs were cultured in DMEM (high glucose) supplemented with 10 ng/mL fibroblast growth factor 4 for 2 days, and then induced. The treatment of BMSCs with differentiation medium was performed in order to induce differentiation, and then they divided into three experimental groups: the HPF group, HGF group and HPF–HGF group. The HPF group, HGF group and HPF–HGF group were cultured in basic differentiation DMEM (high glucose), supplemented with 1 μM HPF, 20 ng/mL HGF, 1 μM HPF and 20 ng/mL HGF. The medium was changed every 3 days. Cells were collected after 7, 14 and 21 days of culture for subsequent experiments.

### 4.7. Western Blot Analysis

The cells in the culture plate were extracted with the configured RIPA (BL504A, Biosharp, Beijing, China) cell lysate for protein extraction. The gel was transferred to a PVDF (IPVH00010, Millipore, Burlington, MA, USA) membrane after electrophoresis, and the PVDF membrane was placed in 3% BSA (4240GR025, Biofroxx, Shanghai, China) and closed at room temperature for 1 h. Afterwards, the primary antibody was added and incubated overnight at 4 °C. The secondary antibody was added, and the membrane was washed at room temperature for 2 h. Finally, the protein bands were detected in an ECL chemiluminescence instrument.

### 4.8. RT-PCR Analysis

Total RNA was extracted by the Qiagen extraction kit, RNA integrity was determined by formaldehyde denaturing gel electrophoresis, and RNA content and purity were determined by UV spectrophotometry. The cDNA was synthesized using the Clontech Reverse Transcription Kit. An amount of 20 μL of cDNA from the reverse transcription system was diluted to 100 μL, then 20 μL of the reaction system was prepared using 1 μL of upstream primer, 1 μL of downstream primer, 1 μL of cDNA, 7 μL of dd H_2_O, and 10 μL of SYBR fluorescent dye, which were mixed in eight consecutive rows. The reaction system was added to the octet and mixed well, then tested on the machine. The relative gene expression was evaluated using the 2^−ΔΔCT^ method. Experiments were repeated three times. The primers are listed in Table 1.

### 4.9. Immunofluorescence Analysis

BMSCs are induced to differentiate into HLCs, and express the liver marker proteins HNF4A and CYP3A4. The expression of specific proteins in HLCs can be determined by immunofluorescence. The medium in the 6-well plates was aspirated, washed once with PBS, and 1 mL of 4% paraformaldehyde was added to each well to fix the cells for 15 min, followed by 3 washes with PBS for the following procedure. A PBS solution containing 10% fetal bovine serum and 0.2% TritonX-100 was added and closed for 1 h, followed by incubation of the primary antibody at 4 °C overnight. The next day, the corresponding secondary antibody was added and incubated at room temperature for 1 h, followed by staining with DAPI and photographing under a fluorescent microscope.

### 4.10. Periodic Acid Schiff (PAS) Staining

In order to detect the storage of glycogen, on the 21st day of BMSC culture, the cells in the 6-well plate were made into glass slides by the method of frozen sections, the samples were fixed in 95% ethanol overnight, and the glycogen stored in the cells was detected by the method of PAS. The sample was then oxidized with 1% periodate for 5 min, rinsed with distilled water for 5 min, and treated with Schiff’s reagent for 10–15 min. After rinsing with distilled water for 5 min, we redyed the sample with Mayer hematoxylin for 30 s, and observed it under light microscope.

### 4.11. Cell Proliferation Assay

BMSCs at the logarithmic growth stage were collected; the density of cells was adjusted to 5 × 10^4^ cells /mL, each well was 100 μL, and the cells were inoculated on 96-well plates and cultured in an incubator for 24 h. After removal, different concentrations of HPF were replaced, according to the experimental group. The culture plates were placed in the incubator and incubated for 24 h, 48 h and 72 h. A total of 10 μL of CCK-8 solution was added to each well after incubation for the required time. The cells were incubated at 37 °C for 2 h, and the absorbance was measured at 450 nm with an enzyme marker.

### 4.12. Animal and Experimental Design

C57BL/6 mice aged 6–8 weeks were provided by Beijing HFK Biotechnology Co., Ltd. (Beijing, China), and were raised in a specific pathogen-free area under sterile conditions, with temperatures of 18–22 °C, humidity of 40–70%, and a normal diet, with free access to food. After a week of acclimatization, mice (n = 12 in each group) were injected with 50% CCl4 (2 mL/kg) olive oil intraperitoneally in order to induce ALI. The control group mice were injected with equal volumes of PBS. After 12 h of injection of CCl_4_, the mice were divided into four groups: (A) CCl_4_ group: tail vein injection of 100 μL PBS solution; (B) CCl_4_ + HPF group: intraperitoneal injection of HPF (80 mg/kg body weight); (C) CCl_4_ + BMSC group: tail vein injection of 100 μL of a suspension containing 5 × 10^5^ BMSCs; (D) CCl_4_ + BMSCs + HPF group: 100 μL suspension containing 5 × 10^5^ BMSCs were injected intraperitoneally with HPF (80 mg/kg body weight). The mice were killed at 24 h and 72 h after the injection with BMSCs and HPF. Blood samples from the retro-orbital sinus of the mice were collected in heparin-free tubes and placed in room temperature for 1 h. The liver was weighed and divided into two parts. One part was frozen in liquid nitrogen for further analysis, and the other part was fixed in 4% formalin for histological observation.

### 4.13. Calculation of Body Mass, Wet Liver Weight, and Liver Index

After the blood was collected, the mice were killed. We immediately removed the whole liver and placed it in cold PBS. We then wiped away the blood and water on the surface with filter paper to calculate the liver index. Liver Index = Wet Liver Weight (g)/Body Mass (g) × 100%.

### 4.14. Serum AST, ALT, ALP, ALB and TBA

Blood samples were obtained from C57 mice at days 1 and 3 after treatment, and supernatants were obtained after centrifugation. The serum of each group of mice was collected, and the contents of AST, ALT, ALP, ALB and TBA were detected according to the kit’s instructions.

### 4.15. Determination of MDA, SOD and GSH Content

Liver samples from each group of C57 mice were extracted on day 3 after treatment, and liver tissue homogenates were prepared. MDA, SOD and GSH expression levels were detected, following the kit’s instructions.

### 4.16. Hematoxylin and Eosin (H&E) Staining

The liver tissue fixed with 40 g/L paraformaldehyde was subjected to gradient ethanol dehydration, methods to induce xylene transparency, paraffin embedding, continuous sectioning, 60 °C baking for 60 min, dewaxing, hydration, hematoxylin dye staining for 5 min, rinsing with tap water, eosin dye staining for 3 min, hydrochloric acid ethanol differentiation, dehydration, neutral gum sealing, observation and photography under the microscope.

### 4.17. Statistical Analysis

In this study, unless specifically mentioned, all data are expressed as the mean ± standard deviation (S.D.). Statistical analysis was performed using GraphPad prism 9. A comparison between groups was made in the way of paired Student’s *t* tests. A *p*-value <  0.05 was considered statistically significant.

## Figures and Tables

**Table 1:** Sequences of the primers used in RT-qPCR assay.

Target Gene	Forward Primer (5′-3′)	Reverse Primer (5′-3′)
β-actin	TGTCACCAACTGGGACGATA	GGGGTGTTGAAGGTCTCAAA
AFP	GCCATCGAAATGCCAGGACA	CGCGTGTAGCCAATGAGGAA
ALB	GGAAGAGTGGGCACCAAGTG	CACAGACGGTTCAGGATGGC
CK18	ACTCTTGGAGCTGAGACGCA	GAGTTGCTCCATCTGCACCC

## Data Availability

Data is contained within the article.
